# *Candida* and Candidiasis—Opportunism Versus Pathogenicity: A Review of the Virulence Traits

**DOI:** 10.3390/microorganisms8060857

**Published:** 2020-06-06

**Authors:** Cristina Nicoleta Ciurea, Irina-Bianca Kosovski, Anca Delia Mare, Felicia Toma, Ionela Anca Pintea-Simon, Adrian Man

**Affiliations:** 1 Department of Microbiology, George Emil Palade University of Medicine, Pharmacy, Science, and Technology of Târgu Mureș, 540139 Târgu Mureș, Romania; anca.mare@umfst.ro (A.D.M.); felicia.toma@umfst.ro (F.T.); ionela.pintea-simon@umfst.ro (I.A.P.-S.); adrian.man@umfst.ro (A.M.); 2 Doctoral School, George Emil Palade University of Medicine, Pharmacy, Science, and Technology of Târgu Mureș, 540139 Târgu Mureș, Romania; bianca.kosovski@umfst.ro; 3 Department of Physiopathology, George Emil Palade University of Medicine, Pharmacy, Science, and Technology of Târgu Mureș, 540139 Târgu Mureș, Romania

**Keywords:** *Candida* spp., virulence factors, microorganism–host interaction

## Abstract

One of the most important questions in microbiology nowadays, is how apparently harmless, commensal yeasts like *Candida* spp. can cause a rising number of infections. The occurrence of the disease requires firstly the attachment to the host cells, followed by the invasion of the tissue. The adaptability translates into a rapid ability to respond to stress factors, to take up nutrients or to multiply under different conditions. By forming complex intracellular networks such as biofilms, *Candida* spp. become not only more refractive to antifungal therapies but also more prone to cause disease. The inter-microbial interactions can enhance the virulence of a strain. *In vivo*, the fungal cells face a multitude of challenges and, as a result, they develop complex strategies serving one ultimate goal: survival. This review presents the virulence factors of the most important *Candida* spp., contributing to a better understanding of the onset of candidiasis and raising awareness of the highly complex interspecies interactions that can change the outcome of the disease.

## 1. Introduction

From over 8.7 million eukaryotic species identified to the current date, the kingdom of fungi has approximately 611,000 species, making up 7% of all eukaryotic species [1]. At the cellular level, fungi are more related to humans than bacteria and belong to the Eumycota group, as chemoheterotrophic organisms [2]. However, only 600 species of fungi are able to cause infections in humans [1].

The genus *Candida* includes about 150 species, but many species are endosymbionts of humans, causing infections mainly in immunosuppressed hosts. Around 80% of infections are caused by *Candida albicans,* although *Candida non-albicans* infections (*Candida glabrata*, *Candida tropicalis*, *Candida krusei*, *Candida dubliniensis*) are becoming more and more frequent [3].

*C. albicans* is a ubiquitous pathobiotic microorganism, a member of commensal flora, which can cause infections in both healthy and immunocompromised patients. Candidiasis may be superficial (e.g., oral, vaginal, mucocutaneous) or profound (e.g., myocarditis, septicemia) [4]. Approximately 75% of women will have at least one vaginal candidiasis during their lifetime, but only 5% will suffer recurrent infections [5]. Despite the fact that common *C. albicans* infections are easily treatable, systemic infection, frequently of nosocomial nature, have a high mortality rate. In the USA, for example, systemic candidiasis has a mortality of 40% [6]. 

With an increase in the number of invasive medical procedures and with an extending population of immunocompromised patients, *Candida* spp. infections are becoming more and more frequent. *C. albicans* remains a major fungal pathogen, although there is a progressive shift to non-albicans *Candida* spp. (NACS), especially *C. glabrata* and *C. parapsilosis* [7]. *Candida auris*, an emergent multidrug-resistant strain first described in Japan in 2009 [8], causes nosocomial outbreaks in healthcare settings, raising concerns at a global level. *C. auris* has limited treatment options, it is often misidentified in laboratories, and it has high transmissibility between patients, thus requiring the implementation of specific control measures, comparable to those used for limiting MRSA (Methicillin-resistant *Staphylococcus aureus*) and CRE (carbapenem-resistant *Enterobacteriaceae*) infections [9].

The relationship between *Candida* spp. and candidiasis takes into account two important factors: microbial-related factors and host-related factors. An imbalance between these factors is associated with the pathogenicity of *Candida* spp. We will focus on describing the virulence factors that may contribute to the evolution of candidiasis, especially in immune-suppressed patients, but also in immune-competent ones.

## 2. The Virulence of *Candida* spp.

The virulence of a microbial species is a measure of the outcome of the microbe–host interactions, rather than a fixed property of the microorganisms. As opposed to primary pathogens that do not require an injured host to cause disease, opportunistic/facultative pathogens such as *Candida* spp. cause disease mainly in susceptible hosts. The strategies that are used to fight against the natural defense mechanisms of the hosts are highly influenced by the environment. Virulence is not a constant property, as it can be enhanced, lost, and even restored in various circumstances [10]. 

*C. albicans* shows great versatility while transforming from a harmless commensal organism to a pathogen. The expression of surface molecules such as adhesins, biofilm formation, the secretion of hydrolytic enzymes, the ability to change its morphology, and its metabolic adaptability are considered “virulence factors”. By means of such virulence factors, *Candida* spp. can quickly adapt to different host niches and cause infections in patients with risk factors such as prior antibiotic therapy, malignancies or an impaired immune system. 

The occurrence of a disease is a partial win in a long-lasting battle: the pathogen tries to escape the immune response and to survive in different host niches, while the host natural barrier and immune cells are trying to prevent an infection or to limit the damage. From an evolutionary point of view, virulence, especially for opportunistic pathogens, is more of a secondary effect or an accident; commensals can cause disease if there is an imbalance in the host–pathogen interactions. The host’s innate immune system plays a very important role in the antifungal fight. For example, neutrophils, by oxidative mechanisms or by releasing extracellular traps, inhibit *Candida* yeast-to-hyphae conversion. The monocytes and macrophages, together with the complement system, the Toll-like-receptors or the lectins that act synergistically to recognize specific fungal molecular patterns (nucleic acids, mannan, β-glucan), induce the release of pro-inflammatory cytokines and chemokines that modulate the host’s immune response [11]. In order to escape the immune system, *Candida* spp. have developed evasion strategies. For example, the immune system can easily recognize a cell wall polysaccharide called β-glucan, but β-glucan is shielded in the outer mannoprotein layer of the cell wall, so that the cell wall structure can trick the recognition mechanisms of the host [12]. 

### 2.1. Adherence and Invasion of the Host Cells

As a commensal or a pathogen, *Candida* spp. need to first attach to various host cells, and they are doing so with the help of adhesins (proteins localized on the surface of the fungal cells), immobilized ligands, such as integrins or cadherins, or indirectly, with the help of other microorganisms. After adherence, the cells have to invade the tissue. The invasion and the damage of the epithelium are mostly considered pathogenic [13] and can occur either by induced endocytosis or by active penetration, according to the type of the host cell. For example, the invasion of the oral cells by *C. albicans* requires the usage of both endocytosis and active penetration, while the invasion of intestinal cells can be done exclusively via active penetration [14]. 

The adherence of the yeast cells to the host surface takes place with the help of adhesion proteins such as als1-7, als9, hwp1 [15], eap1 [16], and pga1 [17]. The ALS (agglutinin-like sequence) family, identified in *C. albicans,* is associated with adhesion and invasion on the host cells, as well as with its iron acquisition strategy. The ALS gene family encodes eight cell-wall proteins (Als1-7 and Als9) that are known to promote adhesion to the epithelial cells. The common opportunistic yeast strains express genes that encode adhesin proteins, but also transporters, lipases, and oligopeptides [18]. Besides this, highly pathogenic strains express genes associated with filamentous growth, biofilm formation, and cell wall structure. Hydrophobicity influences the interaction between Als proteins and abiotic materials, contributing to biofilm formation, for example, on prosthetic devices [19].

Als3 is a surface protein found in *C. albicans* hyphae that mediates the attachment of the yeast cells to endothelial and epithelial cells, as well as to the extracellular matrix proteins. By binding to cell receptors such as E-cadherin and N-cadherin, Als3 induces the endocytosis of the microorganism and, by binding to host cells’ ferritin, it enables *C. albicans* to use ferritin as an iron source [20]. Als3 plays a key role, not only in induced endocytosis, but also in active penetration of the tissue [19], and it is considered a promising target for vaccines [20]. 

Additionally, Eap1, a glucan-cross-linked cell wall protein, glycosylphosphatidylinositol-anchored, which is encoded by the EAP1 gene, plays a role both *in vitro* and *in vivo*, in adherence and biofilm development [16]. Pga1, on the other hand, is a 133 aminoacid GPI-anchored protein that is necessary not only for adhesion and biofilm formation but also for maintaining the cell wall integrity [17].

In contrast with *C. albicans*, *C. glabrata* relies on Epa (Epithelial Adhesins) and Epa-like proteins for attachment to host cell, adhesins encoded by EPA, a subtelomeric gene family. Epa1 plays a key role in the attachment to epithelial cells, and it is characterized by high heterogenicity. As the heterogeneity of this adhesin increases, so do the chances for the occurrence of hypervirulent strains [20]. Epa6 and Epa7 are essential for urinary tract infections. The expression of EPA genes depends on the external concentration of nicotinic acid (NA) [21]. 

*C. parapsilosis* has five ALS genes, as well as six genes for Pga30 (predicted glycophosphatidylinositol-anchored protein 30), that probably help the adherence process, but further research is required for NCAS [22]. It has been reported that 1–10% of the *C. parapsilosis* isolates identified thought conventional biochemical tests are indeed *C. metapsilosis* or *C. orthopsilosis.* While *C. orthopsilosis* and *C. parapsilosis* have similar adhesion properties [23], *C. metapsilosis* is less virulent [24].

Before the penetration of the cellular barrier, *C. albicans* secretes a series of hydrolytic enzymes: “Saps”—secreted aspartic proteinases, PL—a phospholipase, “Lip”—a lipase whose role consists of digesting the cell membrane and surface molecules of the invaded cell, as well as fighting against the immune system of the host [25]. Saps have been increasingly studied, as they facilitate the invasion of the host cells and can counteract the host immune system by degrading IgG heavy chains, C3 protein, collagen, and fibronectin. *C. albicans* can secrete extracellular and intracellular aspartic proteases, and individual genes from the SAP family are expressed at different stages of the infections. Saps have different affinities for different cell types. Sap 1 and 2 are more often expressed during vaginal candidiasis, and Sap 4–6 are secreted after the phagocytosis of the *Candida* cells, inside macrophages. 

NACS also secrete Saps, but their proteolytic activity seems to differ in intensity [26]. Compared with *C. albicans*, *C. glabrata* and *C. parapsilosis* express a low Sap activity. Only three SAP genes were reported for *C. parapsilosis* (SAPP1—SAPP1a and SAPP1b, SAPP2, and SAPP3), but while SAPP1 and SAPP2 facilitate the attachment of the yeast cells, modulate the biology of macrophages, and even disrupt the complement cascade, SAPP2 does not have a proven role in virulence [27]. *C. glabrata* has YPS genes that are responsible for Yapsis Secreted Proteases (Yps), enzymes that have numerous similarities with Sap [28]. *C. tropicalis* possesses four SAP genes, and their expression plays a role in the ability of *C. tropicalis* to cause disseminated infections and to fight the macrophages, but the Sap expression in the oral cavity is not associated with increased virulence [22]. 

Phospholipases are a group of enzymes able to hydrolyze ester linkages in glycerophospholipids, thus producing lysis of the cell. *Candida* spp. produce these enzymes at different rates and it has been shown that *C. albicans* strains isolated from blood specimens present increased extracellular phospholipase activity [29].

### 2.2. The Genomic Plasticity of Candida spp.

Genomic variability is very common in *Candida* spp., being one of the factors that offers this microbial group diversity in the capacity of increased virulence, adaptation to novel environments or resistance to antifungal drugs. The presence of hyper-variable subpopulations in natural populations allows rapid adaptation of *Candida* spp. in times of stress [30]. Genome rearrangements by recombination, loss-of-heterozygosity (LoH), copy number variations, the presence of short tandem repeats (STRs) or transposable elements, chromosomal inversions, or mutations such as deletions, insertions or single-nucleotide polymorphisms (SNPs) are means by which *Candida* species are successful in their evolutionary history [31,32]. In pathogenic *Candida* spp., almost all CTG codons are translated as serine instead of leucine, thus masking β-glucan and interfering with host recognition [33]. The genomic plasticity differs among the *Candida* species, the summative features being presented in Table 1. It is clear that *C. albicans* presents both the most complex genome (including virulence genes such as SAP) and the most means of ensuring its genomic diversity, making the microorganism–host interaction unpredictable, depending on the balance between virulence factors/host immune response.

During *Candida* spp. mitosis, the eventual DNA breakings are handled by the specific cellular repairing mechanisms, and the genome integrity is restored by homologous recombination and non-homologous end-joining [44]. The *Candida* genome also undergoes many rearrangements during asexual mitosis. For a long time, *Candida albicans* was considered an asexual species, but more recent studies showed that it is also capable of mating sexually (producing tetrasomic cells) or parasexually (by concerted chromosome loss, turning back the cells to the diploid state, as no true meiosis is present) [31,45]. Haploid, mating-competent *C. albicans* was also described, with lower virulence and fitness [46]. A parasexual cycle commonly leads to aneuploid forms, when the yeast cells acquire an extra chromosomal information, leading to an increased copy number of genes. Aneuploidy may be induced by stress conditions (UV exposure, antifungal drugs) [47,48]. Changes may be produced by different growth conditions, for example, *in vivo* exposure of *Candida* inducing highly variable isolates as compared to its *in vitro* exposure [30]. 

*C. albicans* is naturally a diploid cell with eight heterozygous chromosomes. Nevertheless, heterozygosity is constantly present in this species, but also in *C. dubliniensis*. LoH appears when information from one of the two chromosomal homologues is lost, for example, because of SNPs at every 200–300 base pairs, and is related to variability in virulence and fitness. This does not necessarily mean that heterozygous genes are non-functional, as many genes that present SNPs that encode stop codons are actually functional. LoH could reflect resistance to fluconazole or phenotypic changes (grey colonies with a lower chance of being phagocytized by macrophages and neutrophils and better fitness for colonizing the host) [47,49]. The LoH rate is variable and increases under the stress of external factors such as antifungal drugs or increased temperature [30]. 

Short tandem repeats are DNA sequences with variable length which contribute to genotypic and phenotypic plasticity, being highly prone to mutations compared to nonrepetitive DNA. The number of STRs is higher in *C. albicans* and *C. dubliniensis* than in other yeast species, and they are responsible for the higher genome-wide mutation rate, pathogenesis, and better adaptation [32,50]. The high number of STRs also favors the allelic homologous recombination [44]. Almost half of the open reading frames in the *C. albicans* genome contain tandem repeats. The pathogenesis of *C. albicans* is also dictated by a large number of multigene families that encode a vast series of enzymes and transporters [51]. 

*Candida* spp. contain transposable DNA elements (less than 5% of the genome, with a low copy number) that can move throughout the genome, mostly by copy-paste mechanisms. These participate in chromosomal rearrangements and recombination of transposable elements, acting as gene promoters, inducing gene interruptions, and thus influencing the transcriptional output. *C. albicans* has more transposable elements than other *Candida* species, contributing to its higher genetic plasticity [41]. The activity of the transposable elements increases under stress conditions, especially when the culture is old [52].

### 2.3. The Morphological Plasticity of Candida spp.

*Candida* spp. can undergo a reversible morphologic transition to better penetrate the epithelial barrier of the host (Table 2). The yeast cells can be found in different shapes: unicellular budding yeast cells or filamentous forms such as hyphae or pseudohyphae, each one playing a distinct role in the invasion process of infection [53]. Pseudophyphae are chains of cells that did not undergo cell separation with contractions at the cell-cell junctions, thus lacking internal cross-walls (septa). True hyphae, on the other hand, are divided by septa. Although hyphae formation is considered a virulence factor, the filamentation process starts only after adhesion to the host surface [54].

The morphogenesis represents a virulence factor mainly for two reasons: hyphae formation facilitates the invasion process and helps *C. albicans* to fight back against the immune system. Hyphae pierce the host cells [14] and because they are larger than yeast cells, are harder to kill by the immune system. Moreover, *C. albicans* can kill macrophages. The killing process involves hyphae formation, followed by elongation, stretching, and the piercing of the macrophage cell membrane [55].

Hyphae formation is an example of anisotropic growth in which the cellular volume expands along a polarized axis. The ovoid yeast cells switch into elongated structures called hyphae that can become more than 10 times larger in size than the cell body, in just a few hours. The growth of hyphae is a well-known process involving the distribution of the Golgi complex toward the growing hyphal tip, assembly of septins from the plasma membrane into a ring and then hourglass and double ring architecture, bud growing, and mitosis septin dispersion [57,58]. Switching between shapes will trigger changes in the biochemical processes that take place inside the cells. *C. albicans* is able to modify its cell cycle transcription machinery mostly by phospho-regulation of transcription factors under the control of kinases, thus being able to transform from a harmless commensal to a pathogen [59]. Hyphae formation, the distribution of nucleic material in *C. albicans,* and the ability to organize into biofilm structures are genetically encoded (for example, SPT20 transcription factor) [60]. Changes in the ambient pH, oxygen concentrations, carbon dioxide, and/or glucose concentration cause *the in vivo* filamentation of *C. albicans* [61]. It was suggested that *Candida* isolates with reduced filamentous growth may not express enough hyphal-specific genes (such as ALS3, SAP4, SAP6), thus being hardly recognizable by the immune system [30].

*C. albicans* hyphae secrete candidalysin, a 31-amino-acid peptide toxin that damages the epithelial cells and also regulates the immunostimulatory capacity of the hyphae, by activating AP-1 (activating protein 1), the transcription factor c-Fos, and MKP1 (MAPK phosphatase 1). Candidalysin is obtained from a preprotein (Ecep1) with the help of hexin-like proteinases. Strains without mutations of Arg61 and/or Arg93 are unable to secrete candidalysin and thus their virulence is decreased both *in vitro* and *in vivo* [62]. For triggering the immune response of the host, candidalysin has to bind to EGFR (epidermal growth factor receptor, also known as ErbB1 or Her1), a membrane-bound tyrosine kinase from the ErbB family [63].

Furthermore, Efg1p and Efh1p, APSES proteins encountered in *C. albicans*, can influence the morphogenesis process. Efg1p influences the expression of genes involved in the metabolic activity of the yeast, activating the genes responsible for glycolysis and inhibiting the genes responsible for oxidative metabolism [64].

Chlamydospores are often used to identify *C. albicans* cells and they are believed to ensure their survival under unfavorable conditions (especially in nutrient-poor environments). They were identified only in *C. albicans* and *C. dubliniensis* and are often used for diagnostic purposes, but their role in virulence has not yet been proved [65]. Efg1p plays an essential role in the formation of *C. albicans* chlamydospores [66], spherical cells with thick walls.

*C. glabrata* does not form hyphae, so to colonize or to cause infections, a breach in the natural barriers of the host is needed. Such a breach can be of an accidental or an iatrogenic nature [67]. Interestingly, *C. glabrata* can rely on *C. albicans* hyphae for inducing oropharyngeal candidiasis, as *C. albicans* can trigger the expression of cell wall protein-coding genes such as *EPA8, EPA19, *CAGL0F00181*, AWP2,* and *AWP7* [68].

Although initially *C. auris* was considered incapable of changing its morphology, recent studies have shown a new phenotypic switch system, one in which *C. auris* can switch shapes from a typical yeast to a filamentation-competent (FC) yeast and to filamentous cells. The switch between the yeast and the filamentous shapes is heritable and it is triggered by the passage through a mammalian organism, while the switch from FC to a filamentous shape is non-heritable and is dependent on temperature (the FC phenotype is promoted by high temperatures) [69].

Non-albicans species such as *C. krusei* and *C. glabrata* can also affect the morphogenesis of *C. albicans*, therefore reducing its pathogenicity. *In vitro*, in mixed cultures of *C. albicans* and *C. krusei*, or *C. albicans* and *C. glabrata*, *C. albicans* shows an impaired ability to form hyphae and a decrease in the expression of Hwp1, a gene that encodes hyphal wall protein 1 (HWP1) [69].

In addition, *C. albicans* morphogenesis can be regulated by “quorum sensing molecules” (QSM), molecules that facilitate an intracellular communication process based on the ability of microorganisms to produce, secrete, and respond to certain hormone-like molecules that accumulate in the extracellular medium as the microbiological population is growing [70]. The dimorphic transition of *C. albicans* cells is inhibited by farnesol, a QSM, when cell density is too high.

The QSM seem to have antifungal activity at high concentrations. Tyrosol stimulates the *C. parapsilosis* oxidative stress response and the expression of its efflux pump genes, while it inhibits virulence-related genes, ribosome biogenesis, and the fungal growth rate. Also, tyrosol influences *C. parapsilosis* metabolism [71] and enhances *C. albicans* filamentation [72].

### 2.4. Adaptability

The adaptability of *Candida* spp. is an important virulence determinant, as the fungus has to reproduce, respond to stress factors, and acquire nutrients in an efficient way to survive.

Although *C. albicans* has been mostly considered an asexual fungus, this has been denied by the discovery of a parasexual stage in its reproductive cycle. *C. albicans* can exist as sterile “white cells” and mating-competent “opaque cells” [73]. The two types of cells have the same genome, but they express different genes, and they have different metabolic preferences and different susceptibilities to antifungal drugs [74]. Sexual reproduction is inhibited by an acid pH [75] and it is regulated by transcription factors encoded at MAT (the mating-type) loci [18].

Mating competent/opaque cells communicate with mating incompetent white cells, enhancing the white cells’ adherence and ability to form biofilms. This form of communication is conducted via pheromones [76].

*C. albicans’* response to stress is a factor of virulence, as it permits the yeast cells to survive and adapt. The response to stress is mediated by “heat shock proteins” (Hsps) [15]. The synthesis of Hsps-type proteins allows the survival of microorganisms at high temperatures, in environments with insufficient nutrients or under high oxidative stress conditions [77]. Hsps 90, for example, is involved in the cellular dispersion inside biofilms, in *C. albicans* susceptibility to antifungals drugs [78], as well as in its temperature-dependent morphogenetic transition [79]. Some Hsps-type proteins that are not encountered in humans (e.g., Hsps 21) are potential targets of new antifungal treatments [15]. *C. glabrata,* on the other hand, resists oxidative stress by producing Srx1 (sulfiredoxin) and two peroxiredoxins (Tsa1 and Tsa2). TSA1 and 2 are expressed in the presence of neutrophils [80].

The replication of the yeast cell, even in an immunocompromised organism, requires prompt adaptation to the new environment, an adaptation that requires metabolic flexibility. High metabolic flexibility ensures a more aggressive replication. In this context, the way the microorganism adapts to take and use nutrients from the external environment can be considered a virulence factor.

In healthy individuals, *C. albicans* is part of the intestinal microbiota, competing with other microorganisms for nutrients. In the disseminated stage of infection, the fungus reaches the bloodstream, a relatively high glucose environment, where it can be phagocytized by macrophages and neutrophils [15].

Once phagocytized, *C. albicans* adapts to an environment that is rich in reactive oxygen species, in reactive nitrogen species, and antimicrobial peptides, but poor in nutrients [81]. In the first stage, in the absence of nutrients, *C. albicans* resorts to gluconeogenesis by using lipids and amino acids as potential sources of energy. Subsequently, trying to escape from the phagocytic cells, *C. albicans* uses glycolysis. Genes that encode the adaptation of the yeast inside macrophages and neutrophils do not have known homologs (not even in *Saccharomyces cerevisiae*, a non-pathogenic fungus related to *C. albicans*) [82]. Moreover, *C. albicans* can induce a rise in the extracellular pH by producing and excreting ammonia, a process in which the transcription factor Ahr1p seems to be involved (Ahr1p is also known to play a role in adherence and morphogenesis). The environmental alkalization helps *C. albicans* to escape phagocytosis, by damaging the macrophage phagosome [83].

Carbohydrates are usually the preferred carbon source of many organisms, with most sugars being converted to Glucose-6-Phosphate or Fructose-6-Phosphate before taking part in the glycolysis process. Glycolysis involves the conversion of hexoses into pyruvate, with ATP (adenosine triphosphate) and NADH (nicotinamide adenine dinucleotide) as produced compounds. Subsequently, energy production can be accomplished in two ways: by breathing or by fermentation [84]. *C. albicans* is a facultative aerobic yeast that prefers the oxygenation of carbohydrates, especially glucose, through the breathing process when oxygen is available, saving its ability to use the fermentation process for anaerobiosis [82]. Preference for glucose can be justified because of the 20 genes that encode glucose transporters (HGT1-HGT20) identified in the *C. albicans* genome [85]. *C. albicans* cells have the ability to modify their transcriptome by inhibiting the genes involved in gluconeogenesis, in the Krebs cycle or in the glyoxylate pathway, encouraging glycolysis and fermentation [86]. Other sugars such as fructose may change the outcome of an infection by increasing the generation time of Candida, thus inhibiting its growth rate [87].

In addition, the host expresses nutritional immunity, forcing microorganisms to develop assimilation mechanisms for acquiring the required intracellular levels of transition metals such as iron, zinc, copper, and manganese. Microorganisms need to acquire enough nutrients to grow and cause disease, while avoiding toxicity and, as the host controls both the essentiality and the toxicity of these metals, the nutrient uptake systems can be considered virulence factors.

The Csr1/Zap1 orthologue regulates the zinc-encoding genes in *C. albicans* and *C. dubliniensis,* influencing their virulence. Zinc is the second most encountered metal in human organisms and its usage by the microorganisms is strictly restricted. When zinc is limited, Zap1 amplifies its transcription and it up-regulates the expression of ZRT1, ZRT2, and ZRT3, genes that encode zinc transporters. CaMAC1, Mn-SOD3, and CTR1 help *C. albicans* to adapt to different copper concentrations, while for *C. dubliniensis* and *C. glabrata,* AMT1 and MAC1, respectively, Amt1 and a putative Mac1 homolog serve the same purpose [88].

### 2.5. Biofilms

In microbiology laboratories, strains grow in perfectly clonal lineage, as a single cell forms a colony that can be seen with the naked eye, but in natural environments, microorganisms are often found in “biofilms”. Many fungi of medical importance (e.g., *Candida* spp., *Aspergillus* spp., *Cryptococcus* spp., *Coccidioides* spp., *Trichosporon* spp., *Pneumocystis* spp.) can grow in biofilms in an attempt to withstand the immune system of the host [89].

Biofilms are complex microbial communities that strike in terms of heterogeneity and offer their inhabitants more chances to survive. Biofilm formation involves primarily the proliferation of yeast cells on a contact surface, either of organic nature (host cells) or inorganic (catheters), followed by their filamentation at the top of the biofilm. Subsequently, the yeast cells are engulfed in a self-produced extracellular matrix, forming a pluricellular tridimensional community with emergent properties [90].

In fact, biofilms are some of the oldest “communities”, with fungi and/or bacteria using them before the colonization of humankind. Biofilms often mix different species of fungi (*C. dubliniensis*, *C. kefyr*, *C. tropicalis,* etc.) or even bacteria (*Pseudomonas aeruginosa*, *Staphylococcus epidermidis*, *Klebsiella pneumoniae*), forming so-called “mixed biofilms”.

In mixed biofilms, microorganisms interact according to the principles of “commensalism” or “antagonism”. Commensalism is defined as a form of cohabitation of several species based on favoring the development of a single species without influencing the development of other microorganisms. Antagonism, on the other hand, involves the development of a species to the detriment of the other [91].

Biofilms are clinically significant mainly for two reasons: firstly, because inside a biofilm microorganisms are more refractory to antifungal drugs, and the dose required to eradicate them can exceed the highest therapeutically attainable concentration of antifungals, and secondly, because indwelling medical devices such as intravascular catheters can be colonized with *Candida* spp. and the cells can detach and cause a disseminated form of infection [92]. Biofilms formed on inorganic surfaces are thus an extracorporeal reservoir of *Candida* spp. and they play a key role in the onset of hospital-acquired infections. Being part of a biofilm brings a series of advantages: increased protection against environmental factors, increased nutrient availability, metabolic cooperation between microorganisms, and more chances for the acquisition of new genetic material [29]. The genetic exchange in *C. albicans* biofilms involves cell fusion and mating [89].

Three main theories were posed for explaining why cells living inside biofilms evade host immune clearance: the first one involves immunological silence (biofilms impair immune sensing mechanisms), the second one involves immunological deviation (the immune response is driven into a non-productive avenue), and the third one involves immune resistance (immune attacks that are effective against free-floating cells cannot dissolute biofilms). A study on mice showed that biofilms induce an immune response highly effective against free-floating cells, yet inefficient against biofilms (the immune resistance model) [93]. 

Inside a biofilm, sessile cells of *C. albicans* are less susceptible to antifungal drugs as compared with planktonic cells (free-floating cells), probably because the matrix forms a diffusion barrier that restricts the ability of the drug to penetrate the layers of the biofilm. Because of the matrix, only the superficial layers of the biofilm are in contact with a lethal drug dose [94]. While planktonic cells usually undergo irreversible genetical chances to maintain a drug-resistant phenotype, biofilms provide an inducible resistant phenotype, which relies on cell density and complex regulatory processes [92].

Under a lack of nutrients, in the starvation phase, and under anaerobic conditions, *C. albicans* survives and forms biofilms on human dentine. As expected, the biofilm formation ability during starvation periods is diminished as compared with the one that cells express during exponential or stationary phases, and the biofilms that are formed are more irregular and have a higher surface area/volume ratio and roughness coefficients. This fact shows once again the adaptability of *C. albicans* to withstand harsh environmental conditions [95].

The capacity of microorganisms to form biofilms is influenced by intrinsic factors (expressions of genes that encode adhesion proteins, signaling systems, the organism’s growth dynamic, etc.) or by external factors (the composition of the substratum, shear, temperature, pressure, etc.) [96]. While forming biofilms *in vivo¸ Candida* spp. might use host components such as neutrophils, epithelial cells, proteins involved in inflammation (e.g., myeloperoxidase, alarmin S100-A9, C-reactive protein) or site-specific proteins, but the role these host components play inside a biofilm is still largely unknown [97].

Biofilm formation is a progressive process, organized in several stages: the early phase, the intermediate stage, and the stage of biofilm maturation. The mature biofilm has a more heterogeneous structure in terms of the distribution of yeast cells and extracellular matrix material. The matrix consists mainly of cell wall polysaccharides such as glucose and mannose [98] and its composition is highly influenced by the environment [22].

The biofilms differ between different species and strains. *C. albicans* biofilm is usually formed by two layers: a basal layer of blastospores, covered by hyphae engulfed in a thick matrix, and *C. tropicalis* biofilm appears as a discontinuous compact monolayer with filamentous morphologies. *C. parapsilosis* can form a discontinuous monolayer or multilayer biofilm composed of aggregated blastospores, yeast cells, and pseudohyphae. *C. glabrata* forms a compact monolayer or multilayer biofilm with only blastospores. The matrix composition of NCAS also varies: *C. tropicalis* has a matrix composed of a low level of proteins and carbohydrates, *C. parapsilosis* matrix contains a low protein concentration and a high level of carbohydrates, and *C. glabrata* biofilm matrix has a high protein and carbohydrate composition [22,99].

The ability of cells to form biofilms does not superpose the process of adhesion of the yeast cells to the host tissue, because biofilm formation is a more complex process that involves cell adhesion, cell multiplication, quorum sensing, and morphogenic changes. Multiple virulence factors are thus involved in contracting multicellular networks such as biofilms, and the attachment of the cells to the surface is just the first step in biofilm development.

The interaction between *C. albicans* cells, for example, and the epithelium of the oral cavity is influenced by the presence of saliva. Some authors consider the possibility that proteins that are normally found in the saliva could act as bridging molecules between the hyphae and the epithelial cells and thus, they can facilitate the endocytosis of the yeast cells, but Phan et al. disproved this hypothesis [20]. In vitro studies showed that even dead hyphae can adhere to and are endocytosed by the epithelial cells found in the oral cavity, similar to live hyphae [100].

The relationships that *Candida* spp. establish with other microorganisms or even between different species can enhance the virulence of one strain. These complex interactions are not completely elucidated, but as polymicrobial infections are often more serious, a growing interest in the discovery of the underlying mechanisms of polymicrobial infections is observed.

Often *C. albicans* coexists with other microorganisms inside biofilms. Interestingly, it has been shown that *Enterococcus faecalis* produces bacteriocin EntV, a protein that inhibits *C. albicans* biofilm formation, decreasing its virulence, without affecting the growth or the chemical sensitivity of the strain [101].

*Pseudomonas aeruginosa*, on the other hand, a bacterium often isolated with *C. albicans*, mainly from catheters, does not possess the ability to adhere to yeast cells, but it can adhere to *C. albicans* hyphae. This event leads to the lysis of the hyphae, mainly due to cytotoxic molecules or degrative enzymes that accumulate in the bacterial foci that are formed on the hyphae [102].

*Streptococcus gordonii*, an oral streptococcus which is part of the viridans group, can congregate with *C. albicans* by using adhesin-receptor interactions. Antigen I/II family adhesins, SspA and SspB, present on the surface of *S. gordonii,* serve as adhesins, and Als3p, a hyphal protein, serves as a receptor for SsspB. A synergistic partnership is formed between the two microorganisms, *C. albicans* being able to enhance the streptococcal biofilm formation, while *S. gordonii* stimulates the filamentation of the fungal cells [103].

It has also been discovered that *Lactobacillus* strains secrete anti-*Candida* factors. *In vitro*, LCS (*Lactobacillus* derived compound) of *Lactobacillus crispatus* and *Lactobacillus fermentum* inhibits the growth of *C. albicans*. Neutral and acidic LCSs inhibit hyphal formation, affecting the pathogenicity. Neutral LCSs downregulate the expression of ECE1, SAP5, ALS3, and HWP1, genes known to be important for the hypha formation [104].

In mixed biofilms where *C. albicans* and *S. aureus* coexist, farnesol (exogenously supplemented or secreted by *C. albicans*) enhances *S. aureus* resistance to antimicrobial drugs by inducing a general stress response. The reactive oxygen species that accumulate intracellularly in *S. aureus* trigger the upregulation of efflux pumps [105]. *S. aureus* uses *C. albicans* hyphae to facilitate its invasion across mucosal barriers. It has been proven that *S. aureus* adheres strongly to hyphae that express the Als3p receptor, while the adherence to hyphae which lack the receptor *als3*Δ/Δ is minimum. The co-penetration of *S. aureus* and *C. albicans* hyphae into the subepithelium is dependent on Als3p expression [106].

Also, *Candida* spp. (*C. glabrata*, *C. tropicalis*, *C. dubliniensis*, *C. krusei,* and *C. parapsilosis)* that can raise the extracellular pH level can enhance the production of staphylococcal alpha-toxin by activating the *agr* (accessory gene regulatory) *quorum* sensing system [107].

Interspecies interactions seem to be highly complex. *Clostridium difficile*, for example, is a strictly anaerobic bacteria living in the gut, an environment in which a facultative anaerobic fungus such as *C. albicans* can be also encountered. *C. albicans* can allow *C. difficile* to survive under aerobic conditions, which, normally, would be toxic for the latter, while *C. difficile* inhibits hyphae formation in *C. albicans,* implicitly affecting biofilm formation, probably through the secretion of p-cresol, a fermentation product of tyrosine [108]. A competitive interaction during biofilm formation was also found between *C. albicans* and non-albicans species such as *C. krusei* and *C. glabrata*. In animal models, infections caused by *C. albicans* alone proved to be more harmful to the tested mice than mixed infections with non-albicans species [109]. On the other hand, some cell wall proteins found in *C. glabrata* (coded by genes such as EPA8, EPA9, *CAGL0F00181*, AWP2, and AWP7) mediate adhesion of *C. albicans,* while Als1 and Als3, proteins found in the hyphal wall of *C. albicans,* are essential for the adhesion of *C. glabrata* [68].

To cause infection, the cells have to detach from biofilm to disseminate to other host niches and, although biofilms consist of a mixture of shapes (yeast, hyphae, and pseudohyphae), the dispersed cells are mainly in the yeast form, and the effectiveness of the dispersion depends on the carbon sources and the pH of the environment [109]. The cells that are released from *C. albicans* biofilm to generate novel communities in different locations possess an enhanced adhesion to the endothelium and overall increased pathogenicity [110].

### 2.6. Age-Related Changes and Candida spp. Virulence

Aging is a complex, multifactorial process that comes with numerous changes in the physiology of a cell, and yeasts make no exception. One of the most important questions is whether aging is a final step in a developmental strategy or simply the result of cellular and molecular damage accumulated over time. It is intuitive to assume that aging and death serve no purpose in the virulence of *Candida* spp., but this is true only if we consider yeast cells solitary entities. *In vivo*, yeast cells are rarely found alone, and virulence is not a characteristic of a single cell, but a characteristic of a population. Yeast cells do not have a predefined life span and their longevity is influenced by their diet or, more specifically, by their diet-induced metabolic capacity and organelle organization [111]. Of course, a longer lifespan seems desirable, but in harsh environments, adaptability proves to be more important. For example, restriction of glucose increases stress resistance, and in the presence of glucose, *C. albicans* prefers respiration to fermentation (Crabtree negative fungus), unlike other yeasts [112]. The lifespan of hyphal and yeast form cells is similar, finite, but is high when they are young and decreases as they grow old. The division capacity of the old cells is diminished due to their progression to senescence, as proved by glycogen accumulation, protein damage or DNA breaks [52,113]. The morphology, ploidy, and DNA content of yeast cells also vary with aging; haploid, diploid, and tetraploid cells become bigger and bigger, and the DNA content of haploid cells increases as incubation time passes [52].

Under a lack of nutrients, or as a response to oxidative stress, intracellular acidification, molecules (plant defensins, polyunsaturated fatty acids, human lactoferrin, farnesol), or in the presence of antifungal drugs such as amphotericin B, caspofungin, miconazole, chronologically aged *Candida* cells can choose to die. This cellular suicide is called apoptosis and it has adaptive value, as the dying cells release substances that help their probably younger and fitter clones to survive. Apoptosis is present especially in the yeast form, but also in the hyphal forms (though in a lesser amount), even if organized in biofilms [112]. The remaining cells live longer and adapt more easily. Aged cells are able to survive longer, but postponing death is not advantageous in the long run, neither for a single cell nor for a population. By choosing to die earlier (the apoptosis of the yeast cells is an active process, not an unavoidable event), they improve the overall virulence of a population [114,115]. Based on these considerations, programmed cell death can play a significant role in virulence.

The age of the host can also influence the outcome of an infection. Aging comes with altered cell functions, which for humans also translates into an impaired immune system. Elderly patients have multiple underlying diseases and an overall increased predisposition for opportunistic infections. Candidemia, in this age group, has a higher mortality rate [116].

## 3. Conclusions

The onset of an infection is dependent on numerous interactions: between different cells of the same population, between different species of the same genus, and pathogen–host interactions. The virulence is not a fixed property of a microorganism, as the environment can trigger the expression of virulence genes, thus changing the outcome. More research is needed, especially for *non-albicans* species, as their incidence is increasing, and the *Candida* genus is extremely heteromorphic. It is difficult to try to reply to the question *Candida* spp. and candidiasis: opportunism or pathogenicity?” in a single sentence. The multitude and diversity of factors make this story long, and practitioners often see unpredictable cases, which makes them wonder how the current knowledge can explain the evolution of the disease.

## Figures and Tables

**Table 1:** Genetic features of *Candida* spp. [18,23,31,33,34,35,36,37,38,39,40,41,42,43].

	*C. albicans*	*C. dubliniesis*	*C. glabrata*	*C. parapsilosis*	*C. tropicalis*	*C. krusei*
Pathogenicity (Intensity)	*+++*	+	++	++	++	+
Ploidy	haploid, diploid, tetraploid, aneuploid	diploid	haploid	diploid	diploid	diploid, triploid
Mating	Sexual, parasexual	Parasexual	No	No	Parasexual	heterothallic
Haploid genome size (Mbp)	14.5	14.6	12.3	13	14.5	10.9
Number of chromosomes	8	8	13	14	5-6	5
Total coding genes	6277	5860	5294	5837	6254	4949
Pseudo-genes	1	123	17	26	1	none
SAP genes	10	8	none	3	4	none
ALS genes	8	3	none	5	16 ALS-like	none
LoH frequency	high	low	none	low	low	common (one large region)
CTG glade	yes	no	no	yes	yes	no
Rate of SNPs	0.3–1.1%	0.008–0.2%	0.01%	0.01%	0.17%	0.30%
Proportion of STRs in the genome	1–2%	1–2%	none	none	none	none
Number of transposable elements	>250 kbp	>250 kbp	none	~160 kbp	>250 kbp	none

**Table 2:** The morphological plasticity of *Candida* spp.

	Morphogenesis	Hyphae Formation	Pseudohyphae Formation	Germ Tube Production
*C. albicans/ C. dubliniensis*	True polymorphism	Yes	Yes	Yes
*C. glabrata*	Lacks polymorphism; exists only as blastoconidia (yeast form)	No	No	No
*C. parapsilosis*	Large and curved pseudohyphae (‘giant cells’) [56]	No	Yes	No
*C. tropicalis*	Polymorphism under special conditions	Yes	Yes/No	No
